# Measuring social, environmental and health inequalities using deep learning and street imagery

**DOI:** 10.1038/s41598-019-42036-w

**Published:** 2019-04-18

**Authors:** Esra Suel, John W. Polak, James E. Bennett, Majid Ezzati

**Affiliations:** 1 School of Public Health, Imperial College London, London, UK; 2 MRC-PHE Centre for Environment and Health, Imperial College London, London, UK; 3 Urban Systems Laboratory, Imperial College London, London, SW7 2AZ United Kingdom; 4 Centre for Transport Studies, Department of Civil and Environmental Engineering, Imperial College London, London, SW7 2AZ United Kingdom; 5 WHO Collaborating Centre on NCD Surveillance and Epidemiology, Imperial College London, London, UK

**Keywords:** Environmental social sciences, Mathematics and computing

## Abstract

Cities are home to an increasing majority of the world’s population. Currently, it is difficult to track social, economic, environmental and health outcomes in cities with high spatial and temporal resolution, needed to evaluate policies regarding urban inequalities. We applied a deep learning approach to street images for measuring spatial distributions of income, education, unemployment, housing, living environment, health and crime. Our model predicts different outcomes directly from raw images without extracting intermediate user-defined features. To evaluate the performance of the approach, we first trained neural networks on a subset of images from London using ground truth data at high spatial resolution from official statistics. We then compared how trained networks separated the best-off from worst-off deciles for different outcomes in images not used in training. The best performance was achieved for quality of the living environment and mean income. Allocation was least successful for crime and self-reported health (but not objectively measured health). We also evaluated how networks trained in London predict outcomes three other major cities in the UK: Birmingham, Manchester, and Leeds. The transferability analysis showed that networks trained in London, fine-tuned with only 1% of images in other cities, achieved performances similar to ones from trained on data from target cities themselves. Our findings demonstrate that street imagery has the potential complement traditional survey-based and administrative data sources for high-resolution urban surveillance to measure inequalities and monitor the impacts of policies that aim to address them.

## Introduction

An estimated 4.2 billion people, accounting for 55% of the world’s population, live in cities^1^. In Europe, 552 million urbanites account for 75% of the total population^1^. Urban populations typically have higher average economic status and education and better average health compared to rural residents^2–4^. At the same time, there are substantial inequalities within cities in income, education, living and neighbourhood environment, health and safety^2,3,5–8^. Inequalities are especially large in large cities like London, where poor and rich live side by side with massive differences in their living environment and health^8–11^. Reducing inequalities is at the forefront of global sustainable development agenda^12^, and is a policy objective in cities such as London^13,14^.

When considered along different dimensions of wellbeing, including income, health, education, employment, crime, housing, and the living environment, inequalities in cities have complex spatial distributions, with some measures, e.g., income and employment, overlapping spatially, and others, e.g. crime and overcrowding, having different spatial patterns. Urban landscapes and inequalities can also change due to processes like gentrification, urban decline, and changes in cities’ economic activities. Measuring urban inequalities at high spatial and temporal resolution is crucial for informing and evaluating policies. A small number of countries have fully linked datasets^15–17^ which allow real-time measurement of inequalities in health, social, and economic status^18,19^. However, such rich data are rare and in most countries and cities, measurement of urban inequalities poses a significant challenge because data on different social, environmental, and health measures come from varied sources, with different spatial resolutions and frequencies, and are often collected using costly processes.

Large-scale imagery data, which are increasingly available through public, private and crowdsourced data, have the potential to significantly advance how fast, how frequently and how locally we can measure urban features^20–30^. That images can be used to measure cities status and the wellbeing of their residents is premised on a number of phenomena: First, some features of cities and urban life, such as quality of housing and the living environment, have direct visual signals in the form of building materials and disrepair, sources of air and noise pollution and green space^31–35^. Others, like poverty, may be visible because they influence or are related to features like housing and neighbourhood environment, the type of vehicles that people use, or even the type of shops^32,34–38^. Finally, within-city variations in outcomes such as health and crime may be detectable in imagery because of their relations to social and environmental factors, even if their absolute level may be harder to predict^28,29,39–41^. This potential mirrors historical attempts to use visual inspections and signals for systemically inferring socioeconomic and environmental characteristics. Charles Booth, for instance, relied on in-person visual inspections of neighbourhoods, and used measures such as state of streets and homes and residents’ clothing for his work for mapping poverty in London in the1890s^33^.

Street images are being increasingly investigated as a source of information in economics and environmental health using both manual visual inspection techniques and machine learning methods^24–29,36,42^. Other studies have attempted to measure perceived neighbourhood attributes such as safety and social class^24–27^, housing price, crime rates, and population density from street images^42,43^. Each of these studies used a different set of images from different settings and cities, employed different analytical methods, and predicted a different set of outcomes at different spatial scales. There has however not been a comparable assessment of multiple outcomes that are likely or possible to be detectable in imagery, in the same city or country, using the same method, same images, and at the same spatial resolution. The lack of comparable analysis makes it hard to evaluate whether and to what extend we can use imagery for detecting urban inequalities in a comprehensive manner.

Here, we use London to evaluate the feasibility and performance of using publicly available street imagery with deep learning for measuring inequalities in multiple aspects of human wellbeing, including income, health, education, employment, crime, housing, and living environment. In addition to its novel empirical scope – comparable analysis of inequalities in multiple outcomes – our approach makes two methodological innovations: First, we directly predicted outcomes of interest from raw images without extracting intermediate user defined features^44^. The network automatically uses features relevant for the measurement task without the researcher explicitly specifying relevant predefined features (e.g. roof types, trees, vehicles). Hence, our approach eliminates the intermediate step of extracting predefined features, a process that requires separate training on databases that may not be publicly available^42,43^. Deep learning methods that automatically learn relevant features commonly tend to outperform methods based on hand-crafted features in visual recognition tasks^44^ and do not rely on whether and to what extend the researchers’ predefined features are relevant for the task at hand. Second, we investigated whether models trained on London data are transferable to other cities, which in turn indicates the extent to which visual features linked to measures of wellbeing are shared across cities. Evaluation of transferability also shows if networks trained on cities with high-resolution data can be used for surveillance in other cities with poorer data.

### Social, environmental and health outcomes

**Figure 1:**
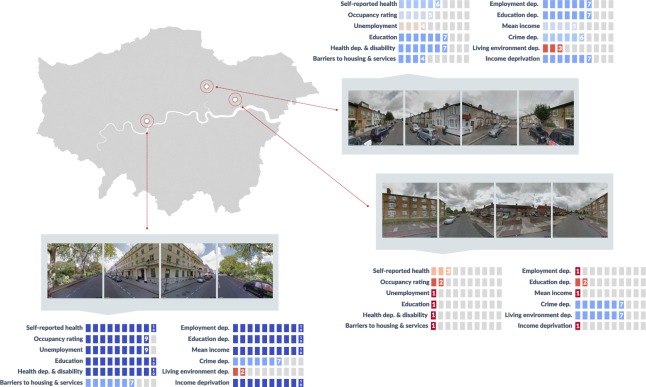
Overview of the street images and outcome (label) data used in the analysis. Four images were obtained for each postcode by specifying the camera direction to cover a 360° view. For each postcode, outcome label data on different wellbeing outcomes were sourced from three public datasets: Census, English Indices of Deprivation, and Greater London Authority household income estimates. In total, twelve different outcomes were used in the analysis (see Fig. 2 for outcomes and Methods for their definition). For each outcome, decile 1 (red) corresponds to the worst-off ten percent of LSOAs, and decile 10 (blue) to the best-off ten percent in London. Outcome labels were assigned to all postcodes within the LSOA. Images courtesy of Google Maps.

We used twelve outcomes for which data for training and testing the network were available from government statistics, for the entire population at a fine scale of Lower Layer Super Output Area (LSOA; average population of 1,614). For each outcome in each city, we calculated deciles of LSOAs, with decile 1 corresponding to the worst-off 10% and decile 10 to the best-off 10% (Fig. 1). The outcomes labels, and their detailed definitions, are listed below. Data on outcomes were from the UK Census^45^ and from English Indices of Deprivation^7^ for those that measure deprivation. The deprivation indices tend to focus on the lower end of distributions of wellbeing outcomes, hence do not capture how LSOAs vary in relation to affluence. The details of how we compute each outcome metric is given below.

- Living environment deprivation: Deprivation index computed using to local air quality estimates, traffic crash rates per 1000 residents, housing in poor condition as defined not meeting the Decent Homes standard in the UK, and houses that lack of central heating as a measure of housing that is expensive to heat. Underlying data comes from Office for National Statistics including housing surveys, UK Air Information Resource, and Department of Transport.
- Mean income: Greater London Authority household mean income estimates (available only for London).
- Income deprivation: Deprivation index relating to low income due to being out-of-work or having low earnings. Underlying data include claimant datasets for government assistance programmes.
- Occupancy rating: Percent of households classified as overcrowded (i.e., having at least one fewer room than required). ONS derives the number of rooms required using a standard formula based on ages of the household members and their relationships to each other.
- Barriers to housing and services: Deprivation index based on physical and financial accessibility of housing and local services. The data used by the ONS to calculate this measure include homelessness, affordability, overcrowding, and distances to schools, supermarkets, health services, and post offices.
- Education: Percent of people who do not have at least a Level 2 education. The five categories for highest attained qualification were: no qualification, Level 1, Level 2, Level 3, and Level 4 and above. Qualification levels in England are defined by the government (where Entry level and Level 1 are the lowest, and Level 8 is the highest).
- Education, [skills, and training] deprivation: Deprivation index related to lack of attainment and skills in the local population both for adults, and for children and young people. The underlying data include education attainment levels, language proficiency indicators, and child benefits claims.
- Health deprivation and disability: Deprivation index based on risk of premature death and impairment through poor physical or mental health. The data used by the ONS to calculate this measure include hospital admissions, and mortality and morbidity rates.
- Self-reported health: Percent of people who reported their own health as very bad or bad; other categories were fair, good, and very good.
- Employment deprivation: Deprivation index based on the adult population involuntarily excluded from the labour market due to unemployment, sickness or disability, or caring responsibilities. The underlying data are from government support claimants.
- Unemployment: Percent of households in which the reference person (determined based on economic activity in priority of order: full time job, part time job, unemployed, retired, other) was recorded as unemployed based on the previous week’s activity. Economically inactive people, those who are not actively looking for work, people who are retired, students, those looking after family or home, or have long-term sickness or disability are not categorized as unemployed.
- Crime deprivation: Deprivation index relating to the risk of personal and material victimisation including violence, burglary, theft, and criminal damage based on police records.

We obtained more than one million images from Google Street View API corresponding to 372,371 postcodes from the four largest urban areas in England: Greater London, West Midlands (including City of Birmingham), Greater Manchester, and West Yorkshire (including City of Leeds). We trained separate networks, described in detail in Methods, to predict each outcome performing ordinal classification from four cut-out images for each postcode using the architecture in Supplementary Information Fig. S1. To evaluate performance of the predictions, we used five-fold cross validation, in which the predicted decile for LSOAs with the known but withheld decile were compared with its actual decile (see Methods for a detailed description).

## Results

### Spatial patterns and inequalities in measures of wellbeing in London

**Figure 2:**
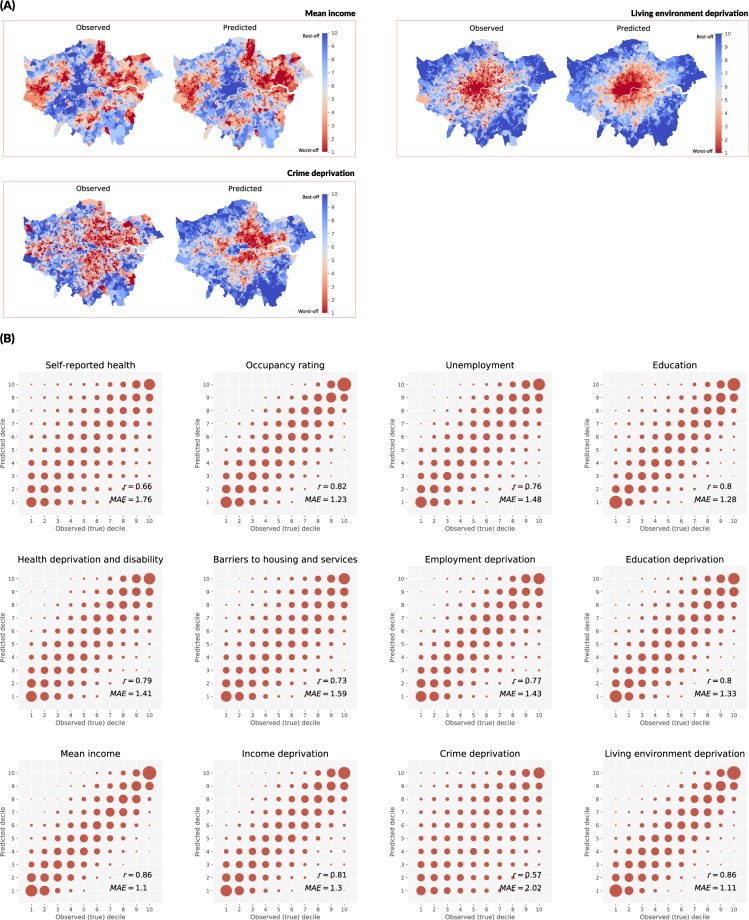
Performance of trained networks in predicting wellbeing outcomes in London: (**A**) Maps of observed and predicted mean income, living environment deprivation, and crime deprivation. (**B**) Comparison of observed and predicted deciles for all measures. In (**B**), each plot displays the number of predicted versus observed decile classes for each outcome, and the size of the circles represent the number of LSOAs corresponding to a specific allocation performance. Perfect allocation would have corresponded to large dots on a diagonal line only, and random allocation to equal-sized evenly covering the entire allocation space. The closer the plot to the former, the better the performance.

Observed data on wellbeing outcomes from government statistics reveal London’s highest income areas are in the city centre and the southwest, in boroughs of City of London, Kensington, Chelsea, and Westminster (Fig. 2A). The poor mostly live in the east, northeast and southeast, as well as in the western outskirts, although London’s east side is being gentrified and contains pockets of well-off areas. Like many other megacities, overcrowding is a problem, especially in the city centre, where space is limited and costly, independent of income^7^. Deprivation in the living environment (an aggregate measure of housing quality, air pollution and road safety) is worse in the inner city, even in wealthier areas, and improves towards the outskirts^7^. There are other complex spatial distributions across the wellbeing outcomes as presented in Fig. 2A and Supplementary Information Fig. S3. For example, income deprivation is correlated with deprivation in employment (r = 0.95) but has only partial overlap with barriers to housing and services (r = 0.68); deprivation in the living environment and crime are weakly correlated with all other measures (average r = 0.28 and r = 0.42 respectively).

### Predictions of wellbeing outcomes

The average Pearson correlation between true and predicted deciles for LSOAs not used in training the network was 0.77 across all outcomes; the average mean absolute error (MAE) was 1.42. The best prediction performance was achieved for living environment deprivation and mean income (r = 0.86, p < 0.01; MAE = 1.11 and 1.10, respectively) followed by occupancy rating, a measure of overcrowding (r = 0.82, p < 0.01; MAE = 1.23). The weakest performance was that of crime deprivation (r = 0.57, p < 0.01; MAE = 2.02) and self-reported health (r = 0.66, p < 0.01; MAE = 1.76) (Table 1).

**Table 1:** Prediction results for multiple wellbeing outcomes: allocation performance for London.

	Accuracy of allocation			MAE	τ	*r*	*k*
	±0	±1	±2				
**Outcome variable**							
Self-reported health	0.223	0.534	0.738	1.76	0.52	0.66	0.47
Occupancy rating	0.307	0.666	0.858	1.23	0.68	0.82	0.63
Unemployment	0.255	0.590	0.800	1.48	0.61	0.76	0.55
Education	0.305	0.668	0.851	1.28	0.67	0.80	0.61
Health deprivation and disability	0.256	0.610	0.820	1.41	0.63	0.79	0.57
Barriers to housing and services	0.233	0.562	0.781	1.59	0.58	0.73	0.52
Employment deprivation	0.256	0.617	0.827	1.43	0.62	0.77	0.57
Education deprivation	0.276	0.640	0.842	1.33	0.65	0.80	0.60
Mean income	0.327	0.717	0.898	1.10	0.72	0.86	0.67
Income deprivation	0.281	0.653	0.854	1.30	0.66	0.81	0.61
Crime deprivation	0.195	0.466	0.673	2.02	0.44	0.57	0.39
Living environment deprivation	0.325	0.708	0.895	1.11	0.72	0.86	0.66

Prediction performances were evaluated using multiple metrics, each column corresponds to a different measure. These measures included accuracy metrics that measures the percentage of correctly predicted classes as well as those that measure the percentage of correctly predicted classes with an allowed error margin of ±1 and ±2 classes, respectively. We also included mean absolute error (MAE), Kendall’s tau rank correlation coefficient (τ), Pearson’s correlation coefficient (r), and Cohen’s kappa coefficient using linear weights (k).

**Figure 3:**
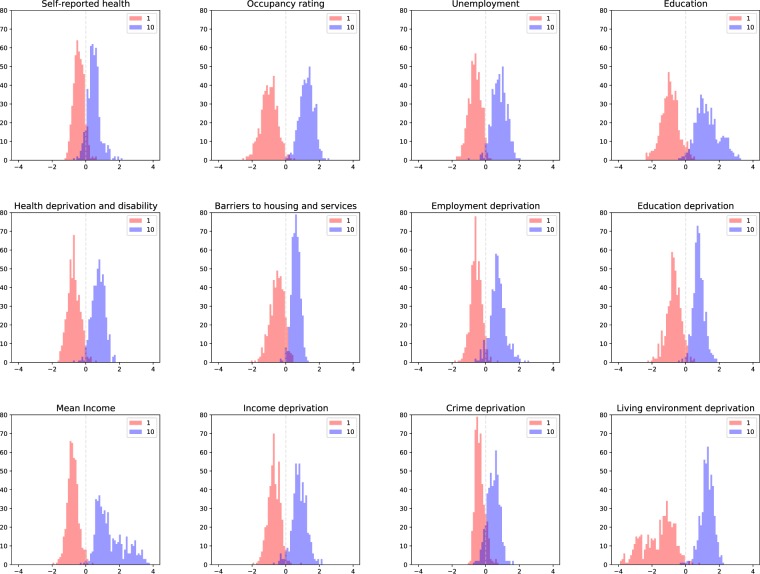
Performance of trained networks in distinguishing best-off and worst-off parts of London. Each cell shows the distribution of the mean continuous output value for each LSOA generated by the final layer of the trained network before conversion to a decile category. Decile 1 (red) corresponds to the worst-off parts and decile 10 (blue) corresponds to the best-off parts in London based on ground truth data. The smaller the overlap of the distributions of predictions for these extreme deciles, the better the network performs in distinguishing them (i.e., measure the full extent of inequalities). Hence, the networks can more easily distinguish between top and bottom deciles for living environment deprivation, mean income, and occupancy than for crime deprivation, self-reported health, and barriers to housing and services.

When considered across the entire range of deciles, an average of 62% (min = 47%, max = 72%) of LSOAs were classified within ±1 of their correct class and 82% (min = 67%, max = 90%) within ±2 (Fig. 2B). In terms of distinguishing the best-off and worst-off LSOAs, which represent the extremes of inequality, there was an average misclassification rate of 4.6% for worst-off and 3.4% for best-off areas (Fig. 3). The predicted best-off and worst-off distributions were highly distinct for living environment deprivation, mean income, and occupancy rating with only 1.5%, 1.9%, 1.7% of worst-off and 0.6%, 0.4%, 0.2% of best-off areas misclassified. In contrast, the predicted best-off and worst-off distributions were partially overlapping for crime deprivation (12.2% and 11.6% misclassification, respectively). These results demonstrate, as expected, misclassification tends to be among neighbouring deciles, which are harder to differentiate (e.g. tell apart images from decile three vs. decile four), but that the network performs well in telling apart the worst-off and best-off areas.

### Transferability of learning

**Figure 4:**
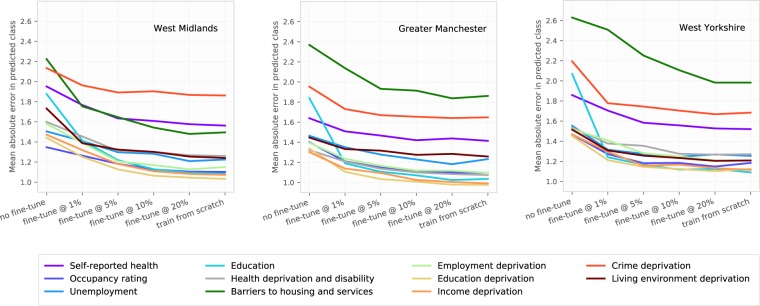
Performance of the network trained on London data applied to images from other cities. The graph shows mean absolute errors for a network trained in London, with network weights fine-tuned using subsets of data from three other target cities. Horizontal axis shows the extent of use of data from target cities, ranging from using the trained network weights from London directly to make predictions in other cities (no fine-tune), to fine-tuning network weights using subsets of target-city data (fine-tune @ 1%, fine-tune @ 5%, etc.), to training from scratch using target-city data only without transferring weights (train from scratch). Allocation performances improve substantially even when only 1% of target city data was used, and similar to performances achieved when training was done from scratch. While there were differences in performance for different outcomes in each city, the top- and bottom-performing outcome groups were broadly consistent.

For evaluating the transferability of learning, we obtained 199,576 images from 53,577 postcodes in West Midlands, 246,056 images from 66,029 postcodes in Greater Manchester, and 212,476 images from 56,277 postcodes in West Yorkshire. When the network trained on London data was used to make predictions in other cities, the average Pearson correlation between true and predicted LSOA classes across all indicators were 0.68,0.71, and 0.66 for West Midlands, Greater Manchester, and West Yorkshire, respectively, and average MAE values were 1.72,1.59, and 1.75, respectively. These correlations were lower, and the errors were larger, than those for London LSOAs. However, when we fine-tuned network weights obtained from London using small sub-sets of data from target cities (using 1%, 5%, 10%, and 20% of all available data for each city), performances improved substantially, and became similar to that of training from scratch using target city data only. For example, when only 1% of target city data was used (corresponding to about 500 postcode locations for each of the cities), average correlation coefficients increased to of 0.78, 0.74, 0.74 and MAE declined to 1.38, 1.49, 1.49 (Fig. 4).

### Strengths and limitations

A key strength of our study is that we apply the same deep learning method for detecting inequalities across multiple outcomes at the same spatial scale and using the same set of images. Therefore, we could test and compare performances for evaluating whether, and for which outcomes, one can use street imagery for measuring urban inequalities. Second, by measuring outcomes from images, our approach eliminated the intermediate step of selecting and extracting predefined features. Nor do we require additional datasets for relating these features to outcomes of interest. Third, evaluation of transferability to other cities can help understand whether street images could potentially serve as low cost surveillance tools in data poor geographies, but will inevitably require further investigation in future work as detailed below.

A limitation of our study was that data used for training the network were available only for those measures for which there are government statistics, and at specific spatial resolutions. Nonetheless, LSOA-level resolution, with an average population of 1,614, is higher than that of most planning activities. Secondly, currently street images are available only at specific times, which may be different from when data used for model training and testing were collected. In our analysis, images and outcome data were a few years apart (see Methods for details). Yet, model performance was high indicating that images contained visual signatures of outcomes measured. Timely and regular datasets, especially in cities that are rapidly changing, can overcome this limitation. Third, image characteristics may be different as a result of seasonal differences, weather conditions and obstructions. Fourth, currently the architectural design of a network is mostly based on intuition, experience and empirical evaluations^46^, which is a common problem in deep learning as it is currently computationally infeasible to search over all possible architectures. Approaches such as ensemble models, used in statistical models for weather and health forecasting with ensembles of statistical models^47,48^, can also be used with deep learning networks if computational efficiency is improved^49^.

### Scalability and future research

Several future research directions arise from our results and from the aforementioned limitations in data and methods. First it would be ideal to extend to the work to other cities and other outcomes. Doing so requires access to street imagery which currently is available through private data providers, with access restrictions or with costs that limit scaling up to many cities. Crowdsourcing images or public sector and governmental efforts for collecting and providing images can enhance opportunities for research and policy and planning purposes. Second, it would be ideal to go beyond street images and use multiple data inputs including from satellites, social media and mobile phones in a single framework which would utilize their complementary information to improve predictions. Doing so requires both data access and development of methods that appropriate and efficiently use multiple sources of data. Third, the transferability of learned networks to other geographies with similar versus different visual characteristics (e.g. from different countries, development trajectories, cultures, and climates) should be further investigated. If transferability is feasible, it will be valuable for training networks in data rich settings and applying them to places with limited data. Investigating transferability, and its features and determinants, will require data on outcomes in diverse settings; increasing transferability requires development of techniques for domain adaptation to overcome differences in visual characteristics. Fourth, transferability should also be investigated over time, to use networks trained for measurement in years after it was trained, and hence reduce the amount of new data that should be collected.

## Discussion and Conclusions

In a comparative, head-to-head analysis multiple social, economic, environmental, and health outcomes, we found that the application of deep learning to street imagery better predicted inequalities in some outcomes (i.e. income, living environment) than others (i.e. crime, self-reported health).

That street imagery could identify variations in the living environment mirrors the intuition that characteristics such as housing quality, pollution (sources), and condition of roads in terms of safety are linked with visual elements, as is the case for occupancy rating which is partly related to population density. Allocation performances were also high for income, because low and high income may have visual correlates in housing, stores, and vehicles^42^. In contrast, the weaker observed performance for crime is consistent with the recent evidence challenging the so-called “broken windows theory” – that visual signs such as broken windows, graffiti, and accumulation of litter may lead to a perception of crime but are not associated with higher rates of crime^50^. The distinction between crime perception and crime rate may explain the higher performances of street imagery when crowd-sourced perceived safety indicators are used^24^ but not for actual crime rates. While performance was low for self-reported health, significantly higher accuracy was observed for the objective measure of health deprivation, which combined data on hospital admissions, and mortality and morbidity rates (r = 0.79, p < 0.01, MAE = 1.41). It is known that self-reported health is affected by differences in health expectation based on socioeconomic attributes^51^.

We also noted that training in one city can be transferred to predictions in other cities in the same country, especially when networks are fined-tuned with as little as 1% of target city images. Fine tuning-supported transferability, if replicated in additional cities and in other countries, may indicate shared visual features of being in deprived and privileged positions. It also means that efficient data-driven urban surveillance could be achieved through libraries of trained networks coupled with a modest amount of local data which can be collected efficiently.

The concentrations of economic activity, innovation, healthcare, education and other public services provides cities with the potential to better the health and wellbeing of their residents. These benefits, however, are distributed with increasing inequality. Realising the consequences of growing inequality for social instability and loss of collective security, many cities are starting to use their resources and powers to confront inequalities^13,14^. To inform and measure the impacts of urban policies for accountability needs reliable and timely data, which is currently restricted by infrequency or low resolution of data from traditional sources. Imagery have the potential to complement traditional survey-based and administrative sources for surveillance at higher temporal and spatial resolution. Street level imagery currently is largely a domain of the private sector (e.g., Google, Baidu) with increasing contribution from crowdsourced platforms such as Mapillary. Such data, while valuable, have inconsistencies and increasingly have access restrictions. Cities have the ability to require or incentivise better access to such data, and use their own assets, e.g., buses and other vehicle fleets which traverse much of the city multiple times, for collecting data, to enhance our ability to measure inequalities and monitor the impacts of policies that aim to address them.

## Methods

### Data sources

#### Image data

We obtained images for London and the next three largest cities in England (West Midlands including Birmingham, Greater Manchester, and West Yorkshire including Leeds) using Google Street View Application Programming Interface (API) (https://developers.google.com/maps/documentation/streetview/intro). We first obtained the Office for National Statistics (ONS) Postcode Directory for the United Kingdom (https://ons.maps.arcgis.com/home/item.html?id=1e4a246b91c34178a55aab047413f29b) and selected the 181,150 postcodes assigned to the 33 local authority districts of the Greater London administrative area, and the 175,883 assigned to the other three cities. For each postcode, API returns, if available, the unique identifier for nearest available panorama image (‘panoid’) most recently taken by Google; the time stamp ranged from 2008 to 2017.

For London, panorama images were available for 156,581 of the postcodes corresponding to 131,465 unique panoid’s. We extracted four image cut-outs for each panorama by specifying the camera direction (i.e., 0°, 90°, 180°, 270°) relative to the Street View vehicle to cover a 360° view. Hence, we obtained a total of 525,860 images corresponding to the 156,581 postcodes in London. For other cities, we obtained 199,576 images from 53,577 postcodes in West Midlands, 246,056 images from 66,029 postcodes in Greater Manchester, and 212,476 images from 56,277 postcodes in West Yorkshire.

#### Outcome (label) data

We obtained data on multiple dimensions of human wellbeing, including income, health, education, employment, crime, housing, and living environment from three publicly available sources: Census 2011 (https://www.ons.gov.uk/census/2011census), English Indices of Deprivation 2015 (https://www.gov.uk/government/statistics/english-indices-of-deprivation-2015), and Greater London Authority household income estimates 2015 (https://data.london.gov.uk/dataset/household-income-estimates-small-areas). All selected outcome data was available at the LSOA level and obtained for the 4,838 LSOAs in London, 1,680 LSOAs in West Midlands, 1,673 LSOAs in Greater Manchester, and 1,388 LSOAs in West Yorkshire.

For each outcome, we calculated deciles of LSOA, with decile 1 corresponding to the worst-off 10% and decile 10 to the best-off 10% in London, and separately for each city in transferability analysis. Individual images collected from postcode locations were matched with LSOA level information as outcome labels.

### Feature extraction using a pre-trained convolutional neural network (CNN)

We used a pre-trained CNN as a fixed feature extractor to convert RGB images to 4096 dimensional codes. We used the VGG16 network^52^ trained with more than 1.3 million images from ImageNet^53^. Street View images were the input to this network with its pre-trained weights, and a 4096-D output from the *fc6* layer was extracted for each image. Therefore, the feature characterisation for each location at this step consisted of four 4096-D vectors, corresponding to the four images per location. Transfer learning, using CNNs pre-trained on large datasets as fixed feature extractors, has been shown to be advantageous to training from scratch especially in scenarios where the target task has access to fewer labelled samples^54^.

### Deep-learning-based assignment to deciles

The assignment of each postcode to an outcome decile is an ordinal classification task, for which we used the network shown in Supplementary Information Fig. S1. As stated above, we used pre-trained weights of the VGG16, and only trained for the weights of the fully connected layers. In this architecture, the network used all four images from each location jointly in the four channels shown in the architecture. The information coming from different channels were aggregated and fed into the final layer, where a single continuous value *p* between 0 and 1 is computed by applying the sigmoid function. Batch normalization^55^ was used in all layers in the network except at the output, where *p* is calculated.

To account for the ordinal relationship among deciles, we used the approach proposed by da Costa and Cardoso^56^, in which the single output *p* value is interpreted as a probability with which Bernoulli trials are performed, i.e. tosses with a coin that has the probability *p* for getting heads and *1-p* for tails, with head and tail corresponding to the events of belonging and not belonging to an output class, respectively. For 10 (ordinal) output decile classes, 10 different coin tosses are considered and corresponding probabilities for obtaining *M* heads are computed for *M* = *1*, …, *10*. This approach casts the ordinal classification problem as a classification task with 10 classes. For training the neural network we optimized the following cross-entropy cost

$$\mathop{\min }\limits_{{\rm{w}}}\sum _{n}\sum _{m}{y}_{n}^{m}\,\mathrm{ln}\,{p}_{n}^{m}$$

where *w* are the network weights, *y*_*n*_ is a label vector for the *n*^th^ sample with a value of 1 for the true label class and 0 for all others, and ${p}_{n}^{m}$ is the probability of the *m*^th^ decile for the *n*^th^ sample. The true labels used for computing the cost function during training were the decile classes associated with the LSOA to which the postcode was assigned to. The network was trained using TensorFlow in Python. We used Adam optimizer^57^ with learning rate 5e-6, and trained the network for 100,000 iterations. The network that yielded the best validation error in the last 5,000 iterations was kept as the final model. We show loss function and mean absolute error in relation to training epochs for both training and validation sets in Supplementary Information (for conciseness we show results only for the mean income; the behaviour was similar for other outcomes).

### Measurement of prediction performance

We used five-fold cross validation. In each fold, 80% of data (i.e., image-outcome pairs for 80% of postcodes) were used for training the network and the remaining 20% were withheld. We then measured how well the trained network uses images to predict outcomes at locations that were not used in training. We repeated this process five times holding out a different 20% of data each time. For prediction, mean continuous output value for each LSOA was computed by averaging postcode-level continuous outputs generated by the final layer of trained networks before the application of the fore-mentioned sigmoid function. The mean value computed for each LSOA was then converted to a decile category, and compared to the LSOA’s actual decile.

We evaluated the performance using Pearson’s correlation coefficient (*r*), Kendall’s tau coefficient (*τ*), Cohen’s kappa (*k*), and mean absolute error (MAE), and accuracy metrics that measures the percentage of correctly predicted classes as well as those that measure the percentage of correctly predicted classes with an allowed error margin of ±1 and ±2 classes, respectively. Full results are shown in Table 1.

### Transferability of trained networks to cities other than London

We evaluated how well a model trained on London data performed in predicting the outcomes of interest using images from other cities. First, we applied the trained network weights from London directly to images from each target city. Second, we fine-tuned the network weights using subsets of postcode level data from each city, specifically using 1%, 5%, 10%, and 20% of the data for fine-tuning the network. Third, we trained networks from scratch for each city using target city data only. The summary of evaluation metrics for each city are provided in the main text.

## Supplementary information

Supplementary Information

## Data Availability

All datasets used in this paper are publicly available and the URLs are provided in the Data Sources section.

## Code Availability

Upon publication, the code will be available at https://github.com/esrasuel/measuring-inequalities-sview and http://equitablehealthycities.org/data-download/.
