# Supplementary material for: Measuring social, environmental and health inequalities using deep learning and street imagery
Source: Sci Rep. 2019 Apr 18;9:6229. doi: 10.1038/s41598-019-42036-w (PMC6473002; doi:10.1038/s41598-019-42036-w)
Supplement: Supplementary file 1 — Supplementary Information [file 41598_2019_42036_MOESM1_ESM.pdf]

Supplementary Information for

# Measuring social, environmental and health inequalities using deep learning and street imagery

Esra Suel*, John W Polak, James E Bennett, Majid Ezzati

*Corresponding author. E-mail: esra.suel@imperial.ac.uk

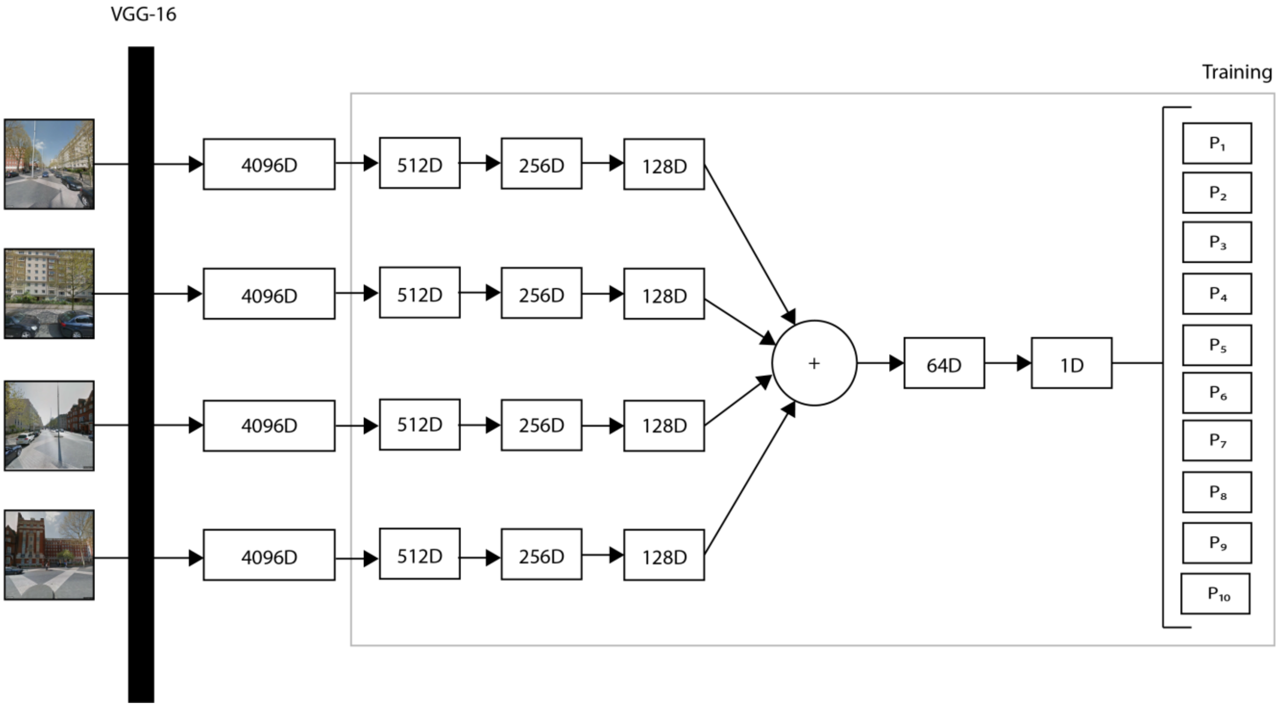


**Figure S.1 The architecture of the ordinal classification network.** Pre-trained weights of the VGG16 were used as is, and we only trained for the weights of the fully connected layers. The network used all four cut-out images from each location jointly, which were then aggregated and fed into the final layer that yielded a single 1D continuous value between 0 and 1 obtained using the sigmoid function. This single continuous value was then used to compute probabilities ($P_1$, $P_2$, … , $P_{10}$) for each of the 10 ordinal decile classes. Images courtesy of Google Maps.

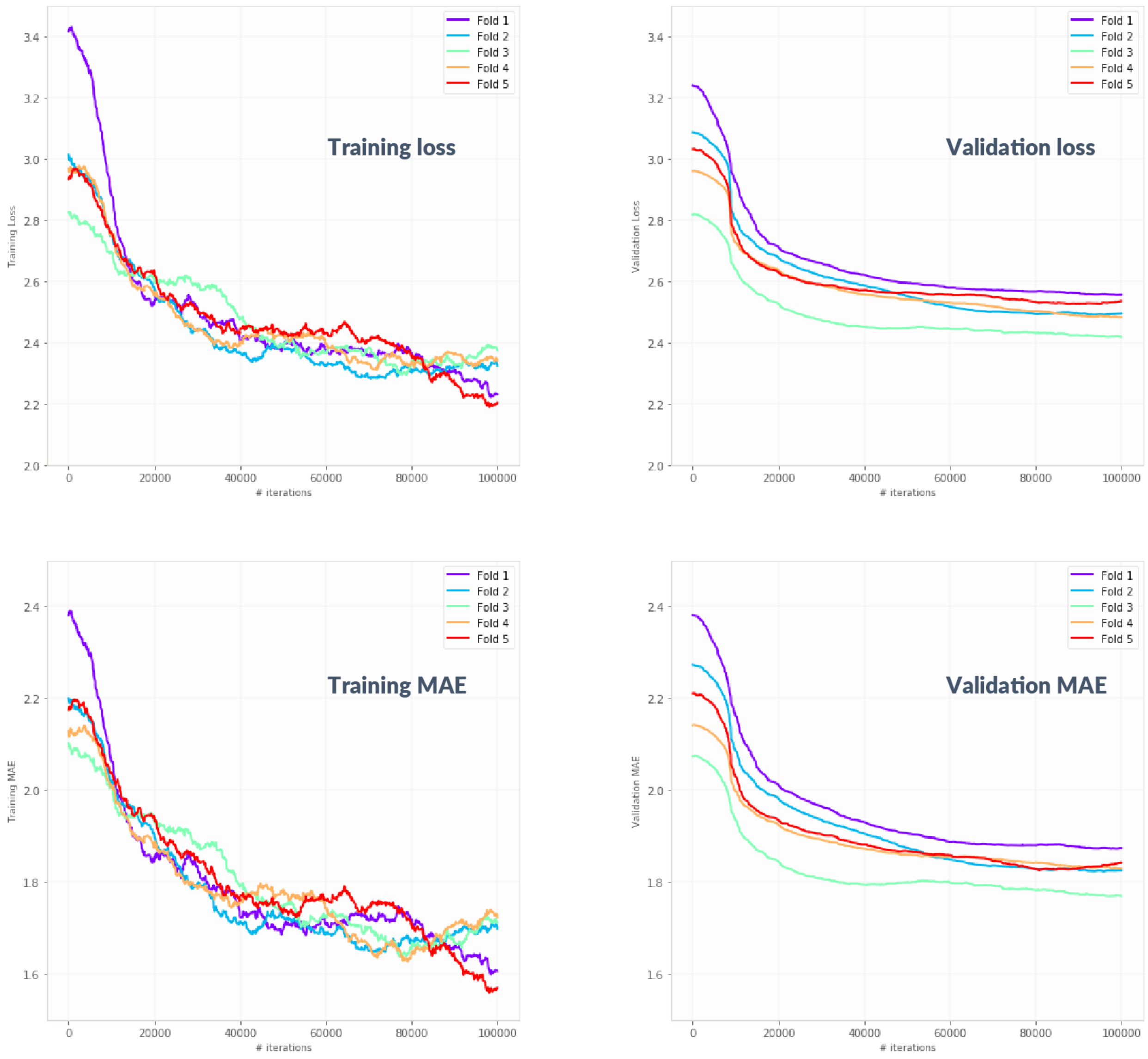


**Figure S.2 Loss function and mean absolute error (MAE) by iteration for training and validation datasets during cross validation.** The five curves in each panel correspond to the 5-folds of cross validation. All results are provided for mean income as an example. The loss both on training and validation sets decrease with increasing number of iterations. The higher variation in the graphs for training data are due to stochastic gradient descent. Note that graphs were smoothed for easier visualisation.

**(a) Self-reported health**

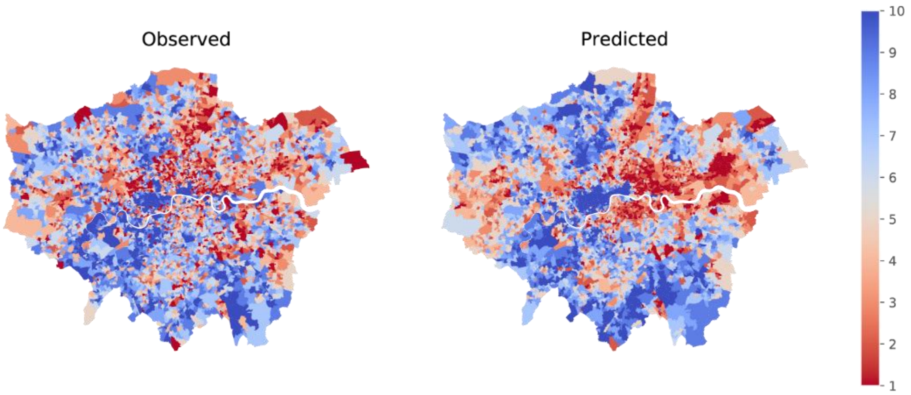


**(b) Occupancy rating**

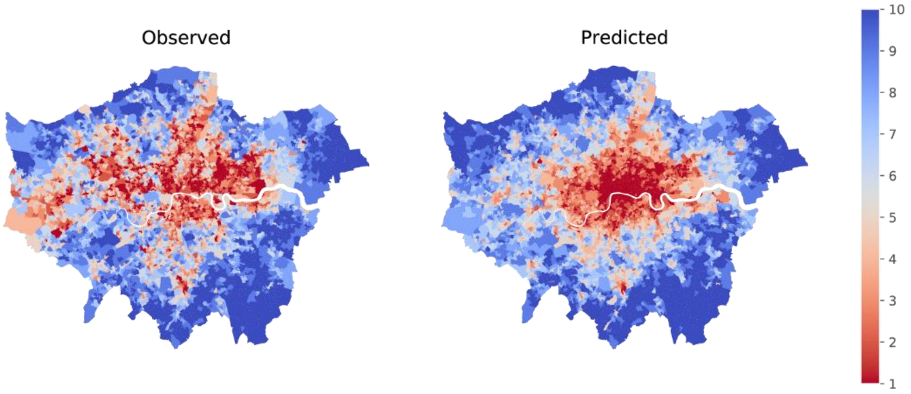


**(c) Unemployment**

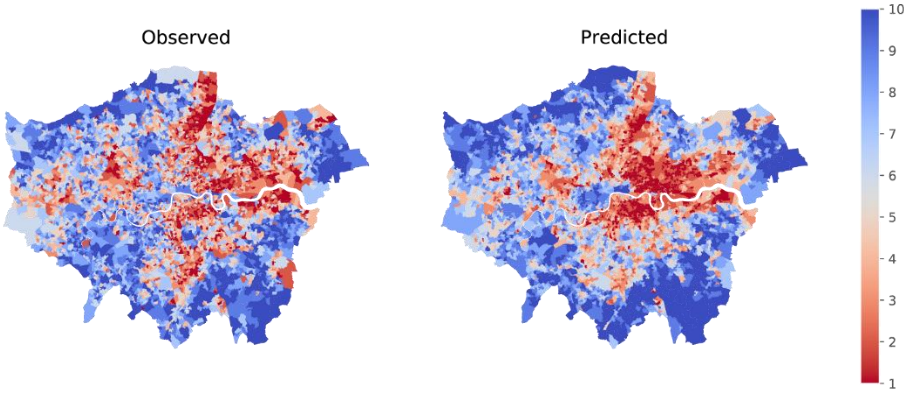

**(d) Education**

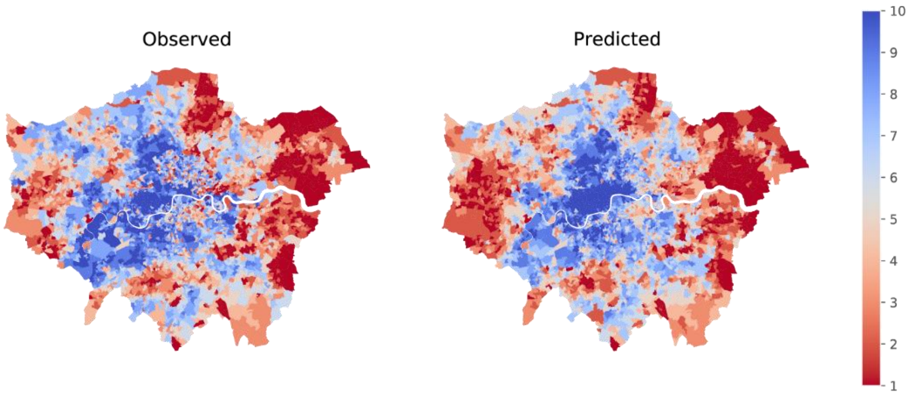


**(e) Health deprivation and disability**

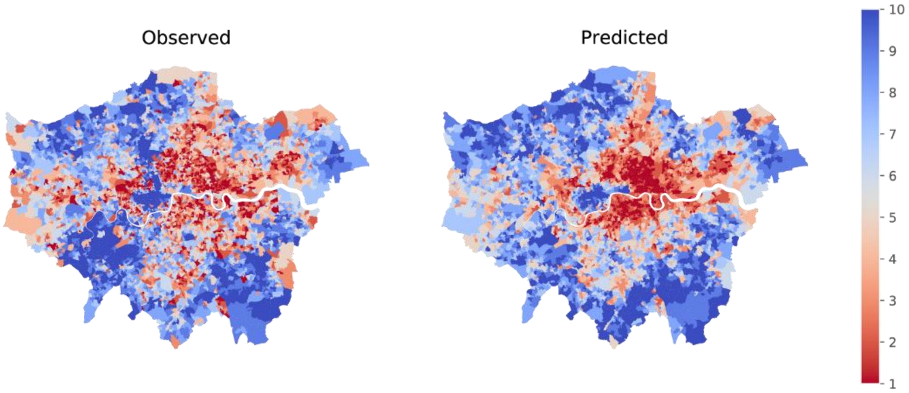


**(f) Barriers to housing and services**

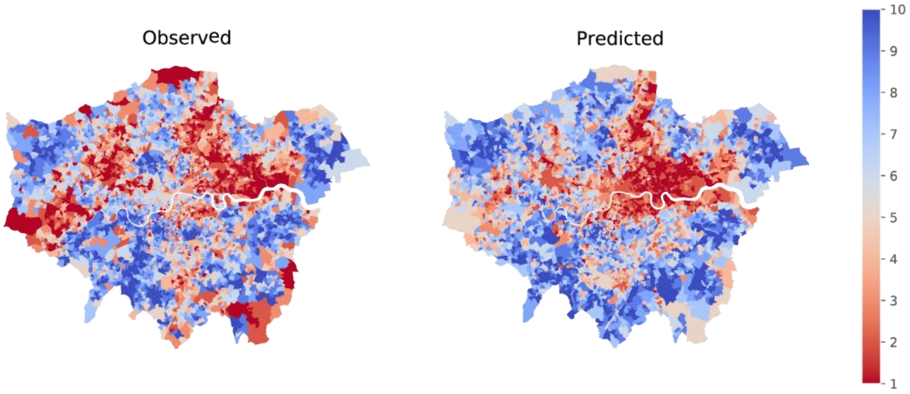

**(g) Employment deprivation**

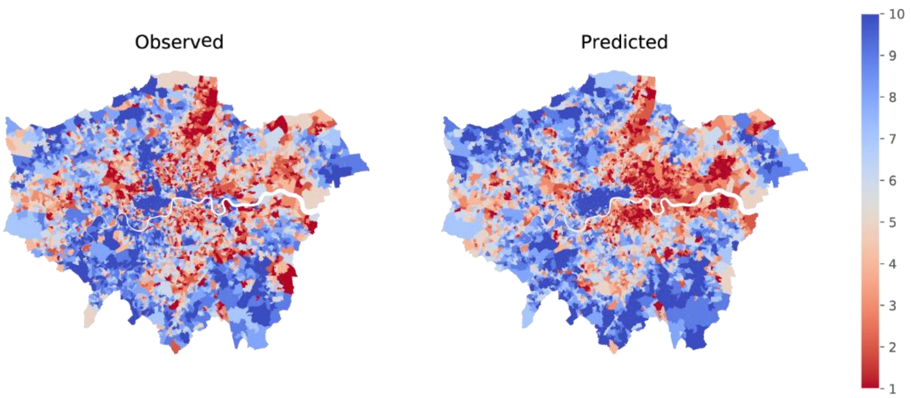


**(h) Education deprivation**

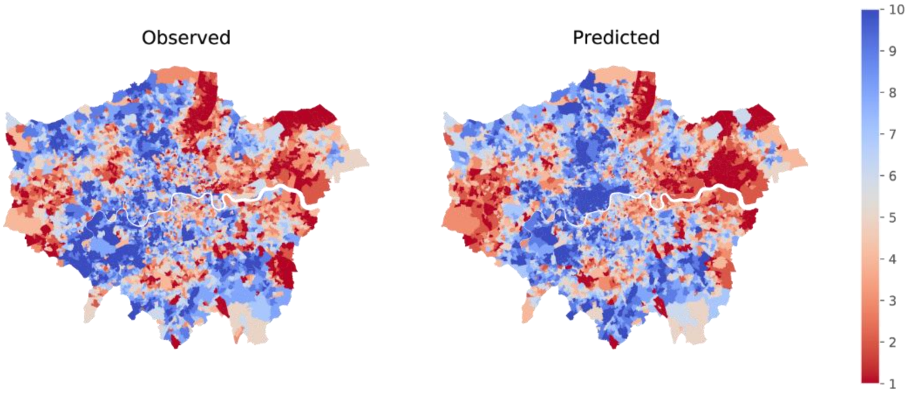


**(i) Income deprivation**

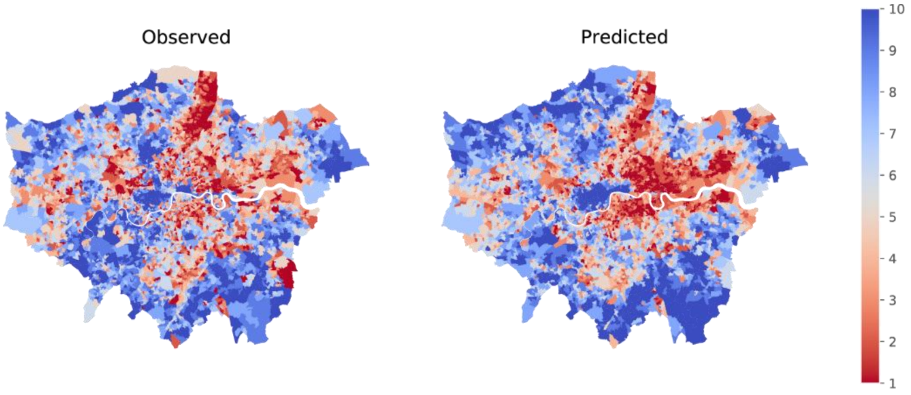


**Figure S.3 Performance of trained networks in predicting wellbeing outcomes in London:** Maps of observed and predicted (a) self-reported health, (b) occupancy rating, (c) unemployment, (d) education, (e) health deprivation and disability, (f) barriers to housing and services, (g) employment deprivation, (h) education deprivation, (i) income deprivation.
